# Use of Endoscopic Scissors to Remove a Foreign Body Impacted in the Proximal Esophagus

**DOI:** 10.31486/toj.24.0117

**Published:** 2025

**Authors:** Muhammad F. Mubarak

**Affiliations:** Division of Gastroenterology, Department of Medicine, College of Medicine, King Saud University, Riyadh, Saudi Arabia

**Keywords:** *Endoscopy*, *esophagus*, *foreign bodies*

## Abstract

**Background:**

The majority of ingested foreign bodies are uneventfully expelled through the gastrointestinal tract. However, anatomically narrowed areas in the proximal gastrointestinal tract create sites of increased retention/impaction of ingested foreign bodies. Foreign body impaction in the esophagus poses a medical emergency because of the complications associated with delayed management: esophageal perforation, infection, and fistula formation. Thus, urgent endoscopic intervention to remove sharp esophageal foreign bodies is necessary.

**Case Report:**

A 45-year-old male presented with a 3-day history of a foreign body in the esophagus. Esophagogastroduodenoscopy identified a horizontally lodged V-shaped fishbone with both lateral edges deeply embedded in the esophageal mucosa. When endoscopic removal using traditional removal accessories failed, endoscopic scissors were used to fracture the spinous process edge of the fishbone, and the fishbone was advanced into the gastric lumen. A makeshift endoscope hood was fashioned from a sterile glove, attached to the distal end of the endoscope, and used to remove the fishbone.

**Conclusion:**

The off-label use of endoscopic scissors to relieve the proximal esophageal obstruction by fracturing the fishbone was integral in achieving successful removal.

## INTRODUCTION

Most ingested foreign bodies are uneventfully expelled via the gastrointestinal tract,^1,2^ but anatomically narrowed areas of the proximal gastrointestinal tract—such as the upper and lower esophageal sphincters and the mid-esophagus at the level of the aortic arch—create sites of increased retention/impaction of ingested foreign bodies.^3^ Similarly, abnormal structural changes of the digestive tract—such as esophageal strictures, webs, or diverticula—pose sites of increased foreign body retention.^4^ Foreign body impaction in the esophagus poses a medical emergency because of the potential complications—including esophageal perforation, infection, and fistula formation—associated with delayed management.^3,4^ The risk of esophageal perforation from foreign body impaction varies depending on several factors, including the duration of impaction and the size and shape of the foreign body.^5,6^ Thus, endoscopic intervention to remove sharp esophageal foreign bodies within 6 hours of clinical presentation is recommended.^7^ Failure of endoscopic removal or complications arising from esophageal foreign bodies necessitate surgical intervention. In a systematic review by Aiolfi et al, surgical intervention was required in 3.4% of all esophageal foreign body presentations, and the overall mortality was 0.85% in patients diagnosed with esophageal foreign bodies.^8^

We present the case of a patient who presented with a 3-day history of foreign body sensation in the throat, odynophagia, and dysphagia. Endoscopy revealed a tightly embedded fishbone in the proximal esophagus.

## CASE REPORT

A 45-year-old male presented to the emergency department (ED) with a history of odynophagia, dysphagia, and the sensation of a foreign body in his throat for 7 hours. The patient reported swallowing a handful of rice cooked with fish that caused him to choke. Because of social embarrassment, the patient force-swallowed the rice. Shortly after, the patient experienced dysphagia and subsequent odynophagia and called a relative (a physician) who instructed the patient to eat a banana and drink a carbonated beverage to help pass any possible impacted esophageal food bolus. When those actions did not provide relief, the patient presented to the ED.

On presentation, the patient's vital signs were stable. Physical examination revealed a middle-aged male in mild distress without drooling, stridor, or shortness of breath. Laboratory workup was notable for leukocytosis 12.9 × 10^9^/L (reference range, 4-11 × 10^9^/L). Otorhinolaryngology consultation was obtained for emergent laryngoscopy. A 70-degree rigid direct laryngoscopy was negative for any foreign body in the oral cavity or hypopharynx. After the laryngoscopy, the patient reported mild improvement and was discharged home with a follow-up appointment in 2 days at the otorhinolaryngology clinic.

**Figure 1.**
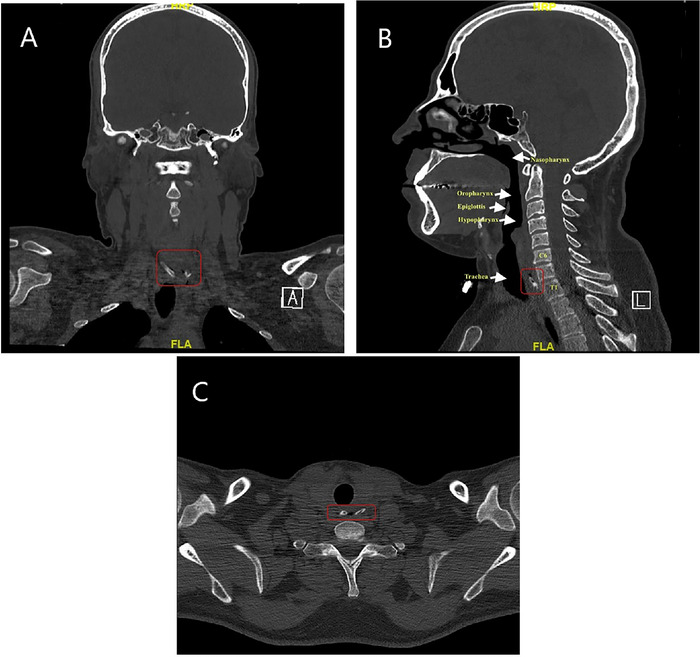
(A) Coronal, (B) sagittal, and (C) cross-sectional computed tomography scans of the head and neck show the impacted foreign body [red boxes].

At clinic follow-up, the patient reported persistent odynophagia, dysphagia, and the sensation of a foreign body in his throat and said he had been able to tolerate only minimal amounts of fluid since symptom onset. Computed tomography (CT) scan of the neck and chest without contrast showed hypopharyngeal mucosal thickening and enhancement. Second-opinion radiologic review of the CT images ascertained the presence of a foreign body in the proximal esophagus (Figure 1). The patient was instructed to return to the ED for an urgent gastroenterological evaluation.

**Figure 2.**
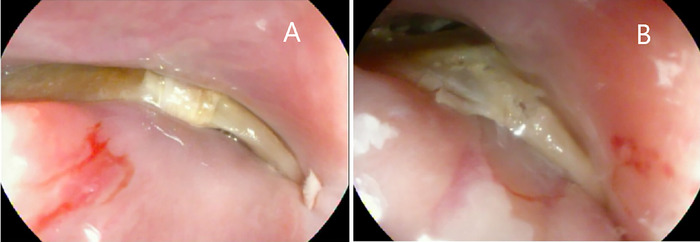
(A) Endoscopic view of the impacted fishbone shows obstruction of the proximal esophageal lumen with both lateral edges embedded in the esophageal mucosa. (B) The spinous process of the fishbone was partially fractured using endoscopic scissors (loop cutter).

At his second ED presentation, the patient had a fever of 38.2 °C and chills. Vital signs were otherwise unremarkable. Updated laboratory workup revealed persistent leukocytosis of 12.7 × 10^9^/L. Emergent esophagogastroduodenoscopy performed in the endoscopy suite with the patient positioned in the left lateral position under general anesthesia with endotracheal intubation was notable for a horizontally lodged V-shaped fishbone 16 cm from the incisors (Figure 2A). The fishbone was obstructing the proximal esophageal lumen with both lateral edges embedded in the esophageal mucosa 2 cm caudad to the upper esophageal sphincter. The edge caudal to the right pyriform sinus was composed of a spiny process that was embedded in but unlikely to be perforating the esophagus, while the contralateral edge had a flat process, and the most cephalad point could not be ascertained endoscopically. The proximal location of the fishbone in the esophagus precluded the use of an endoscopic overtube to facilitate safe endoscopic removal. Probing and manipulation of the fishbone using rat tooth forceps indicated that the fishbone was tightly embedded.

A 13-mm semistiff Captivator polypectomy snare (Boston Scientific Corporation) was used to try to capture the spinous process edge of the fishbone without success. Endoscopic scissors, a one-side-open loop cutter (Olympus Corporation), were then used to fracture the spinous process edge. The endoscopic scissors were applied to the presumed weakest point of the spinous process edge, two-thirds lateral to the V-shaped fishbone vertex. Partial fracture was achieved, resulting in notably decreased horizontal forces and partial release of the spinous process from the lateral esophageal wall (Figure 2B). Further attempts to completely fracture the fishbone were precluded by malfunction of the loop cutter hinge. The tool could not be closed in a scissors fashion onto the fishbone.

**Figure 3.**
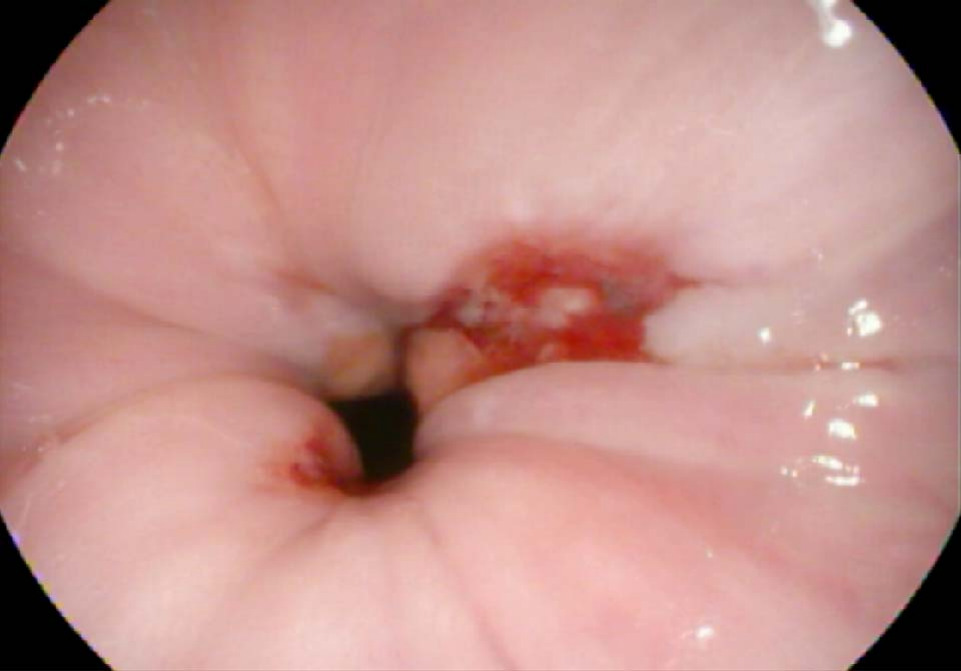
Superficial mucosal tear at the level of the lower esophageal sphincter resulting from endoscopic antegrade displacement of the fishbone.

**Figure 4.**
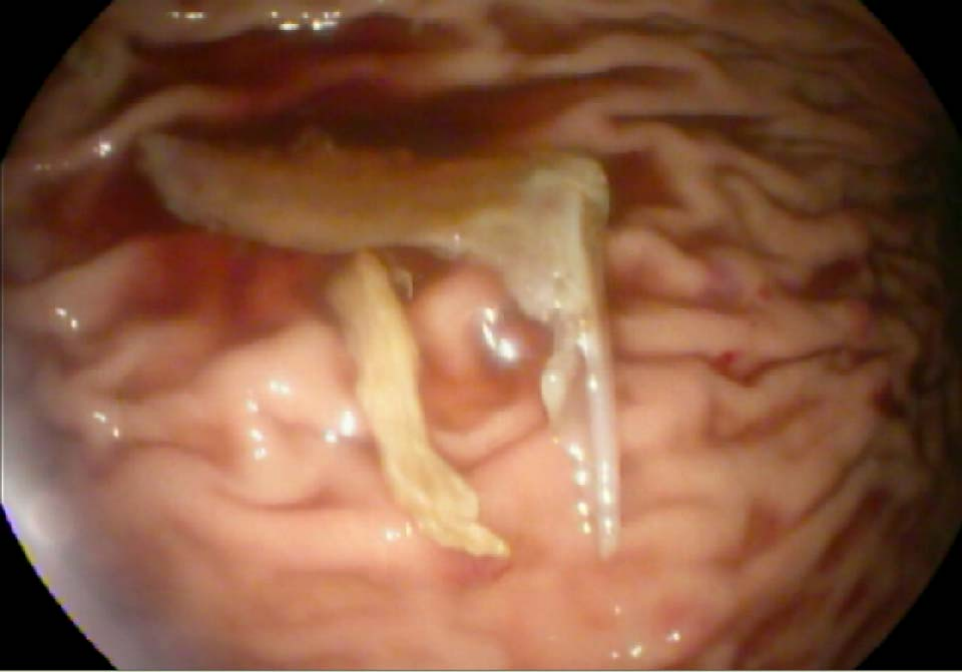
Endoscopic examination of the fishbone in the stomach revealed a complete jawbone with sharp dentition.

Using the rat tooth forceps with gentle and then mild forceful antegrade thrust, the fishbone was dislodged and successfully advanced into the gastric lumen. This action resulted in a 1-cm superficial mucosal tear at the level of the lower esophageal sphincter (Figure 3). Examination of the foreign body in the stomach revealed a complete unilateral lower fish jawbone with raised and sharp dentition (Figure 4).

**Figure 5.**
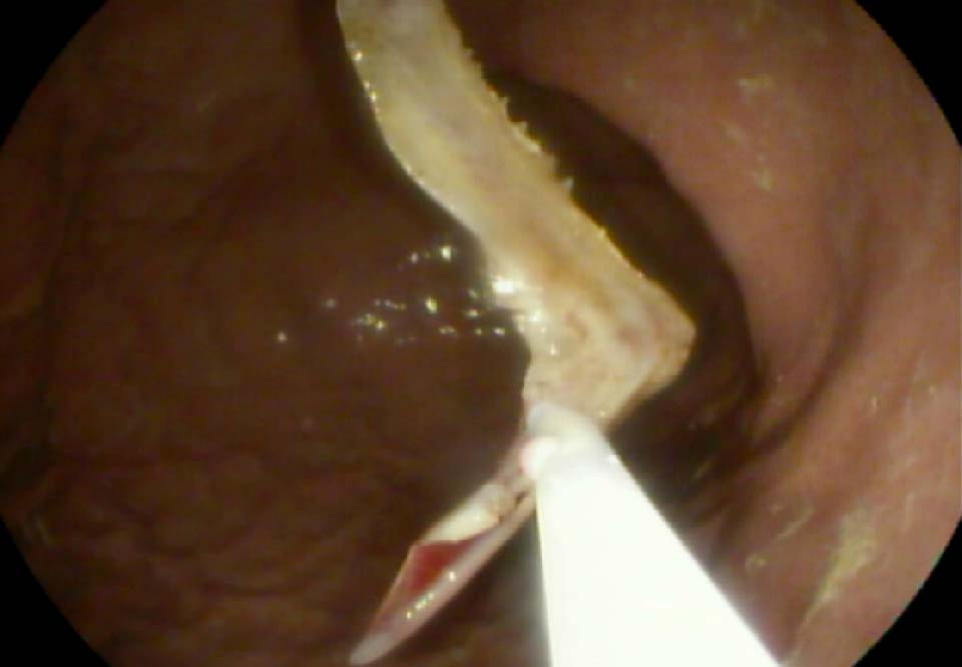
An attempt to further fracture the fishbone in the stomach using the Captivator polypectomy snare (Boston Scientific Corporation) was unsuccessful.

**Figure 6.**
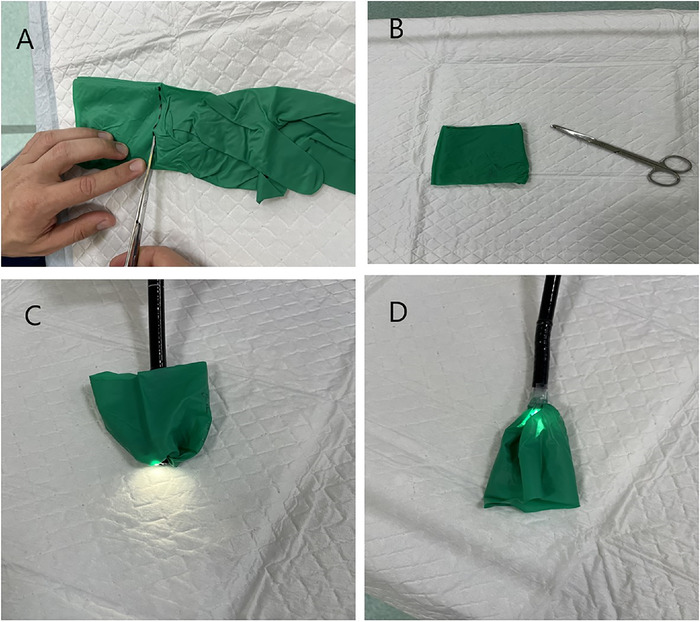
Steps in fashioning a makeshift endoscope hood from a standard 7.5 sterile glove and attaching it to the endoscope. (A) The glove is marked and cut horizontally at wrist level. (B) The makeshift endoscope hood is ready to be mounted onto a standard-sized adult gastroscope. (C) The hood with the flap drawn back is ready for insertion. (D) The image shows the appropriate position of the hood for attempting retrograde retrieval of the foreign body. This position is obtained by withdrawing the hood-mounted endoscope against the lower esophageal sphincter prior to foreign body retrieval.

**Figure 7.**
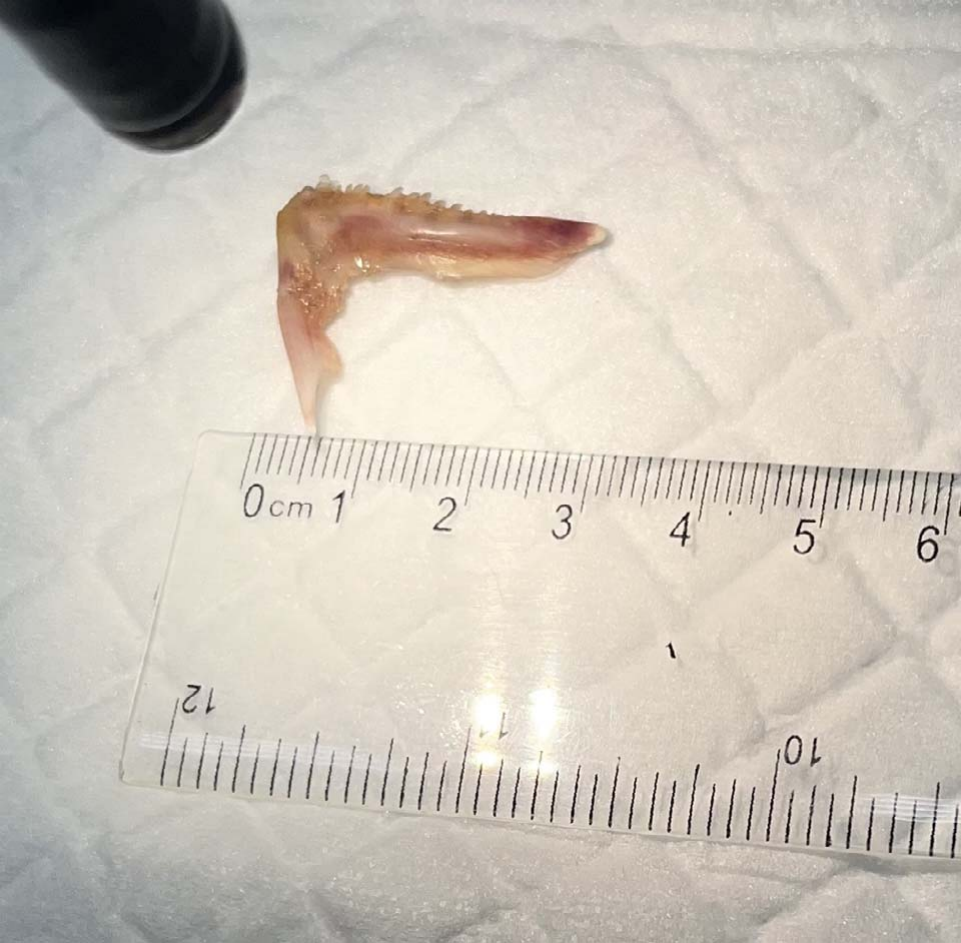
Fishbone after successful removal.

**Figure 8.**
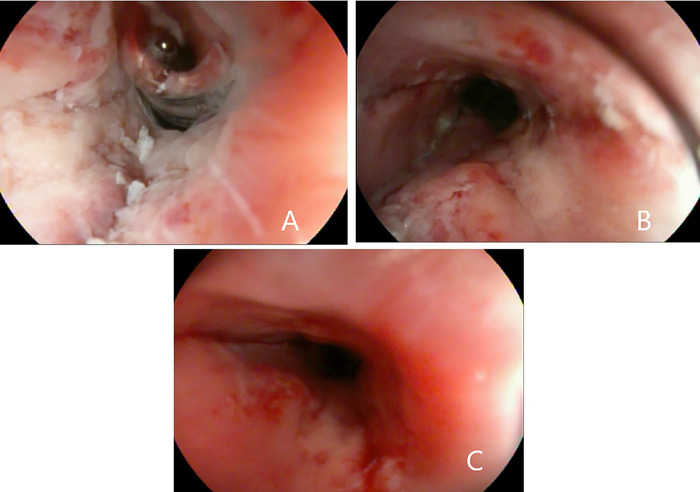
Esophagoscopy findings after fishbone removal. (A) Proximal esophageal mucosal recession and hyperemia of the left lateral wall at the site of the removed fishbone. (B) Proximal esophageal mucosal recession and hyperemia with mucosal tear of the right lateral wall at the site of the removed fishbone. (C) Proximal esophageal mucosal tear with oozing of the right lateral wall at the site of the removed fishbone.

Using the Captivator polypectomy snare in the stomach, further fracture of the spinous process edge was attempted, but the effort was unsuccessful (Figure 5). An overtube could not be used to remove the fishbone from the stomach because of sizing issues. To avoid injuring the lower esophageal sphincter and the remainder of the esophagus upon retrieval of the fishbone, a makeshift endoscope hood was fashioned from a standard size 7.5 sterile glove and attached to the distal end of the endoscope (Figure 6). The endoscope was advanced to the stomach, the fishbone was grasped in a fashion forming an L-shape (with the spinous process forming the horizontal edge to allow for the shortest horizontal diameter), and the fishbone was brought to the distal end of the endoscope within the hood. The endoscope was then removed orally with the fishbone in one piece (Figure 7). Esophagoscopy revealed a moderate bilateral mucosal tear at the site of the fishbone impaction without esophageal muscularis propria exposure (Figure 8).

The patient recovered from the procedure well and was transferred to the medical ward. He was given nothing by mouth for 12 hours and then started on a clear liquid diet and liquid sucralfate 1 g 4 times daily. The patient's leukocytosis and fever resolved. Thirty-six hours from ED presentation, the patient was discharged home after tolerating advancement to full liquid diet. At 1-month follow-up, the patient reported tolerating a regular diet without dysphagia or foreign body sensation.

## DISCUSSION

Early recognition of esophageal foreign body impaction and the identification of the anatomic location based on history taking and radiologic studies are of paramount importance to formulate the best steps for timely management. Complications of delayed management of esophageal foreign body impaction include perforation, infection, major bleeding due to aortoesophageal fistula, mediastinitis, diverticulum formation, fistula formation, aspiration, asphyxia, peritonitis, and death.^3,4,9^

The polypectomy snare, rat tooth forceps, Roth Net retrieval device, biliary stone retrieval basket, and transparent mucosectomy distal cap attachment are endoscopic tools commonly used for foreign body removal.^10,11^ The use of endoscopic scissors for management and removal of impacted foreign bodies in the proximal gastrointestinal tract has also been described in the literature (Table).^2,12-17^

**Table.** Reports of Foreign Body Removal Using Endoscopic Scissors

Study	Number of Patients	Organ(s) Involved	Foreign Body	Technical Success	Endoscopic Scissors Type	Complications
Farr and Pratt, 1989^12^	1	Esophagus	Chicken bone	Yes	Olympus FS-1K (Olympus Corporation)	None
Wilkinson et al, 2011^17^	1	Surgical gastrojejunal anastomosis	Nasogastric tube	Yes	FlexShears (Apollo Endosurgery, Inc)	None
Kee et al, 2014^14^	3	Esophagus, stomach, and duodenum	Fishbone, endoloop placed around the base of a submucosal tumor stalk, and metallic coil used for transarterial embolization	Yes	Not specified (MTW Endoskopie Manufaktur)	None
Park et al, 2015^15^	1	Stomach	Plastic wires	No	Not specified	None
Cohen et al, 2021^2^	1	Stomach	Medical glove	Yes	Ensizor flexible endoscopic scissors, (Apollo Endosurgery, Inc)	None
Su et al, 2021^13^	1	Esophagus	Fishbone	Yes	JHY-FG-23-280-A6 (Jiuhong Medical Instrument Co, Ltd)	None
Duan and Zhou, 2022^16^	1	Stomach and duodenum	Plastic tubes and silk lines	Yes	Not specified	None

In our case, the location of the fishbone in the proximal esophageal lumen and the complete horizontal blockade precluded the use of conventional tools for dislodging foreign bodies. Manipulation of the proximally embedded sharp fishbone presented a high risk for complications. An endoscopic overtube could not be used because the diameter of the widest available overtube was 19.5 mm, too narrow for the approximate 2.5- to 3-cm cross-sectional diameter of the fishbone. The use of endoscopic scissors to fracture the spinous edge of the fishbone effectively decreased the horizontal force responsible for the fixation, allowing for vertical manipulation and eventual dislodgement. The nature of the foreign body became clear once it was in the stomach. The dentulous lower jaw of the ingested fishbone was wide and sharp at its edges, presenting a risk for tear and impaction at the lower esophageal sphincter if retrograde removal was attempted. The fishbone resisted transection in the stomach with the polypectomy snare, likely because of the weakness of the polypectomy snare's plastic sheath. Another type of polypectomy snare such as the LesionHunter Cold Snare (Micro-Tech Endoscopy USA, Inc), which is designed with a metallic tip at the distal end of the sheath, might have provided enough strength to transect the fishbone in the stomach, although such use would be an off-label option. However, the LesionHunter Cold Snare was not available in our endoscopic laboratory. Use of the endoscopic scissors was discontinued after the malfunction in the esophagus. Using a second pair of endoscopic scissors in the stomach was determined to be not cost-effective. The makeshift endoscope hood was effective in minimizing esophageal injury on retrograde removal after the fishbone was aligned to the axis of the endoscope at the least horizontal diameter possible.

No immediate complications occurred despite the patient's delayed clinical presentation prior to endoscopic management. The patient recovered well from his procedure and was clinically ready to be discharged within 36 hours from his second ED presentation.

## CONCLUSION

Sharp foreign body impaction in the esophagus poses a medical emergency necessitating early endoscopic management to avoid complications with a high mortality risk. This case describes the effective use of endoscopic scissors to relieve the proximal esophageal obstruction of a tightly embedded sharp fishbone. The off-label use of endoscopic scissors (the Olympus Corporation loop cutter) to fracture the fishbone was integral in achieving successful removal. However, accessory failure precluded further division of the fishbone. Other studies examining the use of endoscopic scissors for management of proximal gastrointestinal tract foreign bodies are warranted to help identify appropriate accessories, their technical success rates, and their cost-effectiveness.

## Notes

*This article meets the Accreditation Council for Graduate Medical Education and the American Board of Medical Specialties Maintenance of Certification competencies for Patient Care and Medical Knowledge*.
